# Spatio-Temporal Synchronization of Cross Section Based Sensors for High Precision Microscopic Traffic Data Reconstruction

**DOI:** 10.3390/s19143193

**Published:** 2019-07-19

**Authors:** Adrian Fazekas, Markus Oeser

**Affiliations:** Institute for Highway Engineering, RWTH Aachen University, 52074 Aachen, Germany

**Keywords:** sensor synchronization, microscopic traffic data, trajectory reconstruction, expectation maximization, vehicle matching

## Abstract

The next generation of Intelligent Transportation Systems (ITS) will strongly rely on a high level of detail and coverage in traffic data acquisition. Beyond aggregated traffic parameters like the flux, mean speed, and density used in macroscopic traffic analysis, a continuous location estimation of individual vehicles on a microscopic scale will be required. On the infrastructure side, several sensor techniques exist today that are able to record the data of individual vehicles at a cross-section, such as static radar detectors, laser scanners, or computer vision systems. In order to record the position data of individual vehicles over longer sections, the use of multiple sensors along the road with suitable synchronization and data fusion methods could be adopted. This paper presents appropriate methods considering realistic scale and accuracy conditions of the original data acquisition. Datasets consisting of a timestamp and a speed for each individual vehicle are used as input data. As a first step, a closed formulation for a sensor offset estimation algorithm with simultaneous vehicle registration is presented. Based on this initial step, the datasets are fused to reconstruct microscopic traffic data using quintic Beziér curves. With the derived trajectories, the dependency of the results on the accuracy of the individual sensors is thoroughly investigated. This method enhances the usability of common cross-section-based sensors by enabling the deriving of non-linear vehicle trajectories without the necessity of precise prior synchronization.

## 1. Introduction

As the future of road transportation is being shaped around the idea of autonomous mobility, new methods of data acquisition and processing are being developed. Especially in the field of driving assistance systems, there is much current research on detecting and localizing vehicles with many different sensors like radar, LiDAR, computer vision, and acoustics. On the other side, infrastructure-based Intelligent Transportation Systems (ITS) still need further development to exploit the full potential of the already existing sensors. This especially means increasing the level of detail reached with current ITS techniques, which often only deliver aggregated traffic data consisting of datasets with a time resolution of minutes covering traffic parameters like traffic flux (vehicles/hour), density (vehicles/km), and speed (km/hour). This kind of data is a limiting factor for comprehensive analysis on the interaction between individual vehicles. For such detailed analysis, traffic data are required on a microscopic scale, which includes the quasi-continuous trajectory of every vehicle on the road.

The analysis of vehicle-to-vehicle interactions is indispensable for a detailed traffic safety analysis also covering risky situations between vehicles beyond only counting traffic accidents [1]. Many of the surrogate safety indicators rely on such a level of detail [2]. In [3], it was shown that more safety-critical interactions happen when traffic is fluent and vehicle speeds are still at least moderate. In this case, high speed differences lead to more critical time-to-collision values and required deceleration rates to avoid a crash. While analyzing non-fluent traffic can be interesting to calibrate microsimulation models, the lower speeds are less critical from the safety perspective. Beyond safety considerations, efficiency analysis and calibration of traffic flow models can greatly benefit from microscopic traffic data [4]. While these applications rely on the offline processing of the raw data, the work in [5] showed how microscopic traffic models can be integrated into implementing methods for collaborative self-driving vehicles. Similarly, the work in [6] showed how such data can be used to model unmanaged intersections analytically. All these model-based applications could lead to real-time solutions of Vehicle-to-Vehicle (V2V) and Vehicle-to-Infrastructure (V2I) collaboration to support self-driving vehicles in the future.

### 1.1. State-of-the-Art Data Acquisition

Several alternative techniques exist today, capable of acquiring real microscopic traffic data. Of course, one of the most straightforward methods is equipping a large number of vehicles with high-precision Global Navigation Satellite System (GNSS) sensors and recording data from each vehicle [7]. Such methods can also be enhanced by inertial systems [8] to increase positioning accuracy. Other in-car methods can also be used like computer vision systems [9] and fusion of different sensor technologies [10]. While delivering high accuracy and large road coverage, all these techniques share the disadvantage of low traffic coverage. This means that specifically looking at a critical road section of interest and analyzing the dynamics of most vehicles passing through the section is practically impossible. On the other hand, analyzing the dynamics of vehicles specifically equipped with sophisticated sensor systems could be ambiguous, as the behavior of the drivers could be far from natural due to the necessary equipment installed in the vehicles.

A better approach to recording traffic data with a large coverage is the use of infrastructure-based sensors. A very common technique is based on inductive loops [11,12]. They have great reliability and real-time capabilities and are thus widely used for both highway and urban traffic management. Similar to loop detectors, radar sensors work well in various weather and illumination conditions, enabling the detection of individual vehicles and measuring their speeds [13,14]. Other types of static sensors like laser [15], LiDAR [16], and acoustic sensors [17,18] are also capable of detecting vehicles with their respective speeds. Many of the existing commercial products are very easy to use, as they do not need to be installed at great heights. They can be deployed on the ground at the road side, without the necessity of lane closures. This also enables them to be supplied by a battery without the necessity of complicated cabling. The key common drawback of these sensors though is that they gather traffic data from cross-sections, by counting vehicles and measuring their speeds [19]. Thus, the standard use case of these sensors does not enable recording microscopic traffic data over an entire area.

While there are a few studies showing how radar- [20] and laser-based [21] sensors can deliver microscopic traffic data, the most common technique by far is by means of computer vision [22,23,24]. With the appropriate technique of back-projection from pixel coordinates to real-world coordinates, computer vision-based techniques are a cost-effective, non-intrusive, and flexible way of recording microscopic traffic data. Even so, in many cases, the area covered is limited by occlusion, while a dense deployment of cameras is also limited due to the difficulty of road side installation. More specifically, the deployment of surveillance cameras for automatic data acquisition needs thorough planning because they typically need to be deployed at a height where side poles or bridges are required. Installing the cameras at the required height often requires the use of trailer-mounted work platforms and lane closures. Furthermore, the consultation of road administrations, operators of the poles, and sometimes even public transport organizations is necessary. Many cameras additionally require power cabling, with the respective effort and cost, or the installation of batteries on the poles, which can be safety critical. Thus, reducing the number of required cameras by filling gaps for interim sections is of great interest.

An additional difficulty in using already deployed CCTV cameras for computer vision methods is measuring the exact sensor distance or accurately synchronizing the video streams. In our research project “Highly automated tunnel surveillance for catastrophe management and regular operation” (AUTUKAR) regarding automatic video analysis in tunnel surveillance systems, we can only rely on the existing planning documents of the tunnel to determine distances between cameras, which often do not have the desired accuracy. On the other hand, when using real-time video analysis based on video streams, network bandwidth limitation means that a very accurate time synchronization between the streams cannot be guaranteed.

Finally, using cameras is also often restricted by data privacy, a very delicate subject especially in European countries, which often makes application of these sensors impossible. Using sensors that are 100% compliant with privacy laws makes a huge difference in European countries.

### 1.2. Contribution

In this paper, we present a method of spatio-temporal synchronization (offset estimation) of sensors and an appropriate fusion technique to reconstruct microscopic traffic data. The sensors record limited traffic data, which are covered by common detection sensors at fixed cross-sections. Firstly, this results in an enhancement that enables radar and loop detectors to generate area-based microscopic traffic data, even when a precise sensor synchronization is not possible. Secondly, it allows the filling of gaps of data already covering a limited road section, thus reducing the sensor density requirement of computer vision-based systems. The method solves the following problems simultaneously:

- Spatio-temporal offset estimation when measuring the distances between sensors or an exact clock synchronization is not possible
- Vehicle registration without any specific identification like number plates
- Reconstruction of vehicle trajectories with acceleration/deceleration maneuvers only based on cross-section recordings.

### 1.3. Impact

Thus, the presented work has a great impact by enhancing the usability of cross-section-based vehicle detectors by enabling them to acquire microscopic traffic data based only on unsynchronized records of detected timestamps and respective speeds. As many of the cross-section-based sensors do not require recording images or videos, using this method enables the acquisition of vehicle trajectories without any privacy issues. The method also greatly reduces the financial, organizational, and maintenance effort for camera-based acquisition with the ability of filling interim gaps between covered road sections, thus leading to less sensors required to cover a given length. With the proposed method, simple and cost-effective sensors can be used for a detailed safety analysis. If such sensors are deployed along highway exit lanes, this method can be adopted to derive parameters such as individual deceleration rates along the exit lane and to support decision making in regards to appropriate traffic harmonization methods. Furthermore, surrogate safety indicators such as space headways, time-to-collision, or minimal deceleration rates to avoid a crash can be derived, to better understand the evolution of these potential conflicts over time. In addition to presenting the basic principles of the method, we will also analyze the limitations of the method with respect to traffic volume, cross-section distance and detection accuracy.

The remainder of the paper is organized as follows: Section 2 presents the general methodology of the vehicle matching (Section 2.1) and trajectory derivation (Section 2.2). Section 3.1 shows how the underlying data for our experiments have been recorded, while Section 3.2 and Section 3.3 present the experimental validation based on synthetic and real data. In Section 4, we draw the conclusions and discuss potential future research based on our approach.

## 2. Methodology

As formulated in the previous section, we have three different problems to solve: the coordinate systems of the sensor data are not completely synchronized; there is no known matching between individual vehicles; and the dynamic behavior of the vehicles between the two measured cross-sections, which we call gap sections, are unknown. We solved the first two problems simultaneously by making an assumption about the vehicle dynamics, namely that the sensors are close enough and the traffic is fluent enough, so that on average, the drivers make limited deceleration or acceleration maneuvers. This assumption is feasible for the considered orders of magnitude for the distances considered in this work of 100 m–200 m, because in fluent traffic, such sections are covered within several seconds, which restricts the amount of maneuvers. We must also note that it is of great interest to reconstruct microscopic data of fluent traffic as this state leads to high-risk interactions between vehicles due to large speed differences. For non-fluent traffic, analytically determining more complex dynamic behaviors between cross-sections is impossible without the means of driving behavior modeling. Additionally, low traffic speeds also lead to less safety-critical interaction, so from the point of view of traffic data analysis on a microscopic scale, this state is less of an interest than fluent traffic.

We formulate the assumption by describing the vehicles’ movement along the street axis (in one lane) as:

(1) $$s_{i}\left(t\right)=s_{i}\left(t_{0}\right)+v_{i}\left(t_{0}\right)\left(t-t_{0}\right)+a_{i}{\left(t-t_{0}\right)}^{2},$$

where $s_{i}\left(t\right)$ is the position of vehicle *i* at time *t*, $s_{i}\left(t_{0}\right)$ and $v_{i}\left(t_{0}\right)$ are its location and speed at the beginning of the gap section, and $a_{i}$ is its time-constant acceleration.

From Equation (1), it can also easily be derived that:

(2) $$s_{i}\left(t\right)=s_{i}\left(t_{0}\right)+\frac{v_{i}\left(t\right)+v_{i}\left(t_{0}\right)}{2}\left(t-t_{0}\right)$$

so that the position is the same as if the vehicle would have moved with the mean speed between $t_{0}$ and *t*.

### 2.1. Vehicle Matching and Offset Estimation

Based on the assumption formulated above, we treated the vehicle matching as a probability density estimation problem. To solve it, we applied the expectation maximization algorithm described in [25]. The algorithm aims at finding the maximum likelihood estimates from incomplete data by looping over two iterations:

- Estimation step (E-step): Find an estimate for the complete data sufficient statistics.
- Likelihood Maximization step (M-step): Determine the parameters of the distributions based on the estimated data.

**Figure 1:**
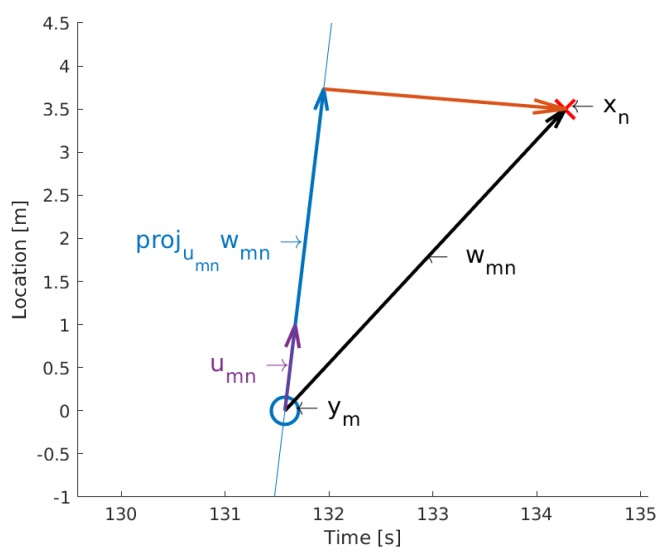
Geometric interpretation of the distance of a data point from the line trajectory of another data point. The data point $y_{m}$ is the time and location of a vehicle detection in the first sensor, while $x_{n}$ is a corresponding data entry from the second sensor. $u_{mn}$ is the unit vector with the gradient calculated as the mean of the two speeds measured at the sensors. $proj_{u_{mn}}w_{mn}$ is the projection of $w_{mn}$ onto this unit vector. The distance of the second data entry from the optimal linear trajectory corresponding to the mean speed can be derived by subtracting the projected vector from $w_{mn}$.

This method was originally intended to determine the parameters of a few statistical distributions based on a larger set of incomplete data. We applied this method to our problem as follows: Suppose we have two sensors detecting vehicles with their time-stamps and speeds at two consecutive cross-sections. The set of *M* vehicles with their respective speeds from the second sensor form linear Gaussian kernels, while the *N* vehicles and speeds from the first sensor form the data upon which we are trying to find the distribution parameters. The mean of the individual Gaussian models is defined by the mean speed between a vehicle pair as a linear trajectory, as defined in (2). The standard deviation is defined as the distance of the data point to the trajectory line. The geometrical interpretation of this distance can be seen in Figure 1, where $y_{m}=\left(t_{m0},s\left(t_{m0}\right)\right)$ is the recorded time and location of a vehicle entry from one sensor. $x_{n}$ is an entry from the dataset of the other sensor. We define the vector $w_{mn}=x_{n}-y_{m}$ and $u_{mn}$ as being the unit vector of the linear vehicle trajectory calculated from the mean speed between the two entries. We calculate the perpendicular vector $w_{mn}^{⊥}$ from the desired linear trajectory to $x_{n}$ as follows:

$$\begin{array}{cc} w_{mn}^{⊥} & =-proj_{u_{mn}}\left(w_{mn}\right)+w_{mn}=-u_{mn}\frac{u_{mn}^{⊺}w_{mn}}{∥u_{mn}∥}+w_{mn}=\\ & =w_{mn}-{\left({\left(u_{mn}^{⊺}w_{mn}\right)}^{⊺}u_{mn}^{⊺}\right)}^{⊺}=w_{mn}-{\left(w_{mn}^{⊺}\left(u_{mn}u_{mn}^{⊺}\right)\right)}^{⊺}=\\ & =w_{mn}-u_{mn}u_{mn}^{⊺}w_{mn}=\left(I-u_{mn}u_{mn}^{⊺}\right)w_{mn} \end{array}$$

Thus, the distance of the point to the trajectory line is given by:

$$\begin{array}{cc} ∥w_{mn}^{⊥}∥ & =∥\left(I-u_{mn}u_{mn}^{⊺}\right)w_{mn}∥=∥\left(I-u_{mn}u_{mn}^{⊺}\right)\left(x_{n}-y_{m}\right)∥ \end{array}$$

The Gaussian model of the vehicle pair (m,n) has the form:

(3) $$p\left(x_{n}|\Theta_{m}\right)=\frac{1}{\sqrt{2\pi \sigma^{2}}}\exp^{-\frac{∥\left(I-u_{\mathrm{mn}}u_{\mathrm{mn}}^{T}\right)\left(x_{n}-y_{m}\right)∥}{2\sigma^{2}}},$$

In the rest of the paper, we will use the notation $U_{mn}=I-u_{\mathrm{mn}}u_{\mathrm{mn}}^{T}$ for the projection matrix used to calculate the perpendicular vectors on the unit vector $u_{mn}$. The Gaussian distribution of all vehicles use the same standard deviation, while they have equal membership probabilities $P\left(m\right)=\frac{1}{M}$. The mixture model is defined as:

(4) $$p\left(x|\Theta \right)=\sum_{m=1}^{M}p\left(m\right)p\left(x|\Theta_{m}\right),$$

while the log likelihood function given the set of model parameters is:

(5) $$ln\left(p\left(x|\Theta \right)\right)=\sum_{n=1}^{N}ln\sum_{m=1}^{M}p\left(m\right)p\left(x_{n}|\Theta_{m}\right).$$

**Figure 2:**
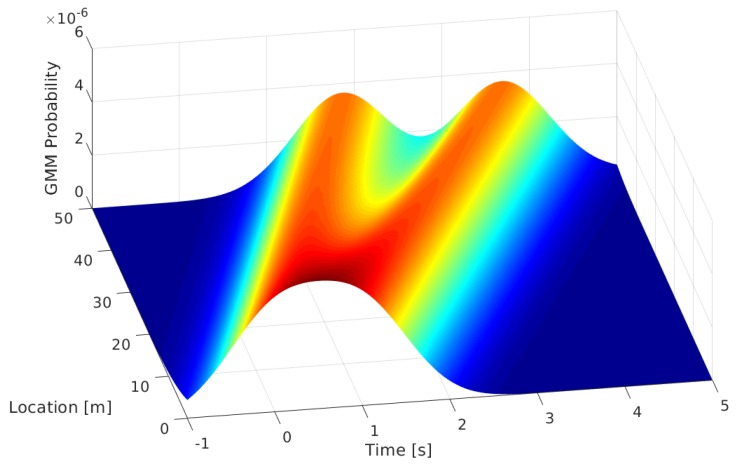
Distribution of a Gaussian mixture model consisting of two linear vehicle trajectories. If there is one data entry *x* with a specific speed and two possible data entries *y*, it results in two projected vectors $proj_{u_{mn}}w_{mn}$ and two Gaussian distributions from Equation (3). The Gaussian distributions depend on the orthogonal distance from the projected vector and add up to the Gaussian mixture model seen in this figure.

Figure 2 shows a probability distribution of a Gaussian mixture model defined by Equation (4) with two vehicle trajectories, with the first one having a higher speed than the second and the vehicles passing the detection sensor with a difference of one second.

As described above, the maximization of this likelihood function is solved by looping through two steps. The first one is calculating the expected complete-data log likelihood function given by:

(6) $$Q\left(\Theta ,\Theta^{old}\right)=\sum_{n=1}^{N}\sum_{m=1}^{M}p\left(m|x_{n},\Theta^{old}\right)ln\left[p\left(m\right)p\left(x_{n}|\Theta_{m}\right)\right].$$

The missing data consist of the soft matching of the detected vehicles so that (6) can be derived by determining the a posteriori probabilities of Gaussian model *m* matching vehicle *n*:

(7) $$p\left(m|x_{n},\Theta^{old}\right)=\frac{p(x_{n}|\Theta^{old})}{\sum_{m=1}^{M}p(x_{n}|\Theta^{old})}=\frac{\exp^{-\frac{∥U_{mn}\left(x_{n}-y_{m}\right)∥}{2\sigma^{2}}}}{\sum_{m=1}^{M}\exp^{-\frac{∥U_{mn}\left(x_{n}-y_{m}\right)∥}{2\sigma^{2}}}}$$

The second step consists of maximizing the *Q* function given by (6) with respect to the Gaussian parameters. At this point, we have to define the set of parameters Θ being considered here for optimization. As already stated above, one of the common parameters is the variance of the Gaussians given by $\sigma^{2}$. As our initial problem was to determine the offset of the sensors, the other parameter is a translation, which moves the Gaussians along the space and/or time axes. As the set of Gaussians is based on vehicle data recorded from one sensor, it is feasible to use a common translation vector t for all the Gaussians, as the offsets have to be consistent for all vehicles recorded within a sensor. After applying the logarithm to the exponential function, we get:

(8) $$\begin{array}{c} Q\left(\Theta ,\Theta^{old}\right)=-Nln\left(M\sqrt{2\pi }\right)-\frac{N}{2}ln\left(\sigma^{2}\right)-\\ -\frac{1}{2\sigma^{2}}\sum_{n=1}^{N}\sum_{m=1}^{M}p\left(m|x_{n},\Theta^{old}\right){∥U_{mn}\left(x_{n}-\left(y_{m}+t\right)\right)∥}^{2} \end{array}$$

Note that in Equation (8), the first term is constant and can be neglected. Taking the partial derivative with respect to the translation parameter t and equating it to zero give:

(9) $$t=U^{-1}(\sum_{n=1}^{N}\sum_{m=1}^{M}p\left(m|x_{n},\Theta^{old}\right)U_{mn}\left(x_{n}-y_{m}\right))),$$

where $U=\sum_{n=1}^{N}\sum_{m=1}^{M}p_{mn}U_{mn}$ and $U^{-1}$ is its inverse matrix. By substituting t back into the function *Q*, we can maximize with respect to σ again by partial derivation and equalizing with zero:

(10) $$\sigma^{2}=\sum_{n=1}^{N}\sum_{m=1}^{M}p\left(m|x_{n},\Theta^{old}\right){∥U_{mn}\left(x_{n}-\hat{y_{m}}\right)∥}^{2},$$

where $\hat{y_{m}}=y_{m}-t$. In accordance with the EM-algorithm, we cycle through the E- and M-step until the change in either the log-likelihood function or in the parameters of the Gaussian models (σ and t) is small enough to consider that the algorithm has converged.

We summarize our approach as an algorithmic description in Algorithm 1. Here, the E-Step consists of recalculating the soft matching probabilities, in other words the probability that vehicle *m* from the first sensor matches vehicle *n* from the second one. The M-step is a spatio-temporal shift of all M vehicles from the second sensor with a common value $\hat{t}$ and an update of the standard deviations of the Gaussians.

**Algorithm 1:** Vehicle registration using expectation maximization.

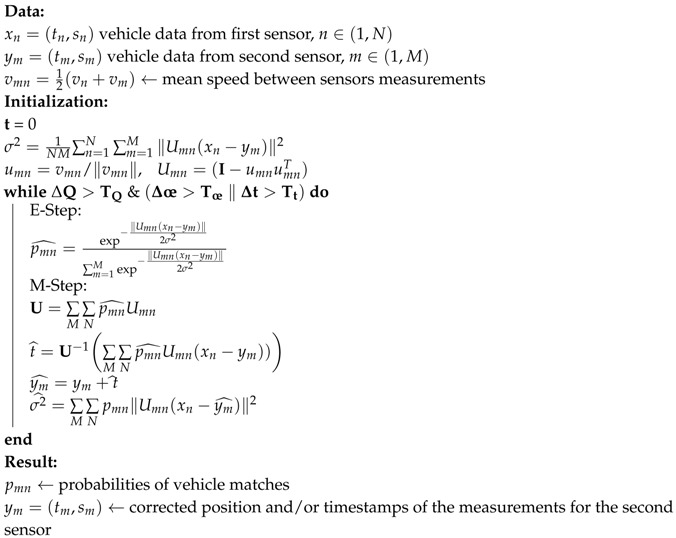



### 2.2. Microscopic Data Reconstruction

In this section, we will take the next step in reproducing microscopic traffic data, based on the results of the previous section. Let us suppose that with the means of the EM-algorithm, we found the spatio-temporal offset of the sensors, and we also have a correspondence matrix P where the entries $p_{mn}$ are the last computed prior distribution of the vehicle matching problem from Algorithm 1. We can compute the matrix M with the entries $r_{mn}=∥U_{mn}\left(x_{n}-y_{m}\right)∥$ being the respective point to line distances.

Now, after we found the sensor offsets and the soft matching probabilities $r_{mn}$, we applied the Hungarian algorithm [26] to find the first correspondences between vehicles. The Hungarian algorithm is a polynomial time solution to the assignment problem of two datasets. We treated the vehicles of the first and second sensor as the two different datasets. The Hungarian algorithm uses a cost value for the assignment, which in our case was the distance from the linear trajectory from the already shifted entries of the second sensor to entries of the first one. Therefore, we used the same $r_{mn}$ values as a cost value in the EM-algorithm and formed a matrix Rwith the number of rows and columns corresponding to the data entries of the first and second sensor. The overall procedure of the Hungarian algorithm is as follows:

- **Matrix reduction**: The cost matrix is reduced row-wise with the respective minimal value of the row. In cases of entire columns greater than zero, a column-wise reduction is also performed.
- **Line covering**: Cover resulting zeros with the minimum number of lines (horizontal and vertical). If the number of zeros, which are unique in the respective row and column, is equal to the smallest of the two matrix dimensions (number of vehicles in first or second sensor), an optimal matching has been found.
- **Additional reduction**: Reduce all non-covered elements by the minimal non-covered value and add that value to all elements covered by both horizontal and vertical lines. Continue with line covering again.

The result of the assignment problem is a set of pairs, where for each vehicle of the first sensor, a vehicle of the second sensor is matched. The assignment is only an interim step towards reconstructing the trajectories as it still assigns the vehicle pairs based on a linear trajectory based on mean speed between the vehicle pairs. However, the assignment is very robust, because the real trajectories of the vehicles are similar enough to the linear ones in order for the matching to be successful.

We also have to note at this point that the resulting assignments also hold assignments of outliers, as they have also been translated according to the EM-algorithm. Thus, both false positives and false negatives in the data of both sensors need to be considered. We applied a threshold of 3σ to the assigned values of $r_{mn}$. We considered all the assignments with costs above this threshold as outliers and removed them from the dataset, as they were misdetections (false positive or negative) of either sensor. A trajectory should not be reconstructed for such singular data entries. One important aspect here is that we have no possibility of identifying a false positive that fits well to a trajectory of a true positive detection. If a sensor delivers a false positive that fits a smoother trajectory than the true matching, that will influence the assignment of the Hungarian algorithm and also the prediction of the resulting trajectories. On the other hand, most of the sensors used in our experiments showed a tendency towards false negatives rather than false positives, so the chance of a false positive detection influencing the results was quite low. We will analyze the effect of false detection more thoroughly in the following section.

After finding appropriate assignments, we reconstructed the trajectory of the vehicles between the two sensors using curves, which can be parametrized to connect the two points while also following the detected speeds profile. In other words, the connecting curves s(t) need to satisfy:

(11) $$s\left(t_{n}\right)=s_{n},\;\;\;\;s\left(t_{m}\right)=s_{m},\;\;\;\;\frac{\delta s}{\delta t}\left(t_{n}\right)=v_{n},\;\;\;\;\frac{\delta s}{\delta t}\left(t_{m}\right)=v_{m}$$

where $\left(t_{n},s_{n}\right)$ and $\left(t_{m},s_{m}\right)$ are the time and positions of the assigned sensor detections, while $v_{n}$ and $v_{m}$ are the respective speeds. The polynomial curves used in this work were Bézier curves [27]. They have very useful properties, being easy to calculate and derive, so acceleration and deceleration rates can easily be analyzed. Thus, the use of Bézier curves enables the acquisition of a relatively high amount of dynamic behavior of road users, given the fact that only simple cross-section data were used and no complex driver behavior models were applied.

The general definition of Bézier curves is:

(12) $$B\left(u\right)=\sum_{i=0}^{n}b_{i}B_{i,n}\left(u\right),$$

where:

(13) $$B_{i,n}\left(u\right)=\left\{\begin{array}{cc} \frac{n!}{(n-i)!i!}{\left(1-u\right)}^{n-i}u^{i} & ,\mathrm{if}\;0\leq i\leq n\\ \;0 & ,\mathrm{otherwise} \end{array}\right.$$

called Bernstein polynomials of degree *n*. $b_{i}$ are the control points that define the curve, and as *u* increases within the interval [0,1], the Bézier curve is drawn from the first towards the last control point. From Equation (12), the general formulation of the $r^{\mathrm{th}}$ derivative of a Bézier curve of degree *n* can be deduced to:

(14) $$B^{\left(r\right)}\left(u\right)=\sum_{i=0}^{n-r}b_{i}^{\left(r\right)}B_{i,n}\left(u\right),$$

where:

(15) bi(r)=n(n−1)⋯(n−r+1)∑j=0r(−1)r−jrjbi+j.

We now proceed to define the control points based on the input data. The requirements defined by Equation (11) ensure that the positions and the speed values are respected by the trajectory. The disadvantage is that there is no continuity in the second derivative. In other words, a vehicle trajectory fulfilling those requirements will have an unrealistic speed change directly at the first and last control points. Thus, we add the continuity requirements of the second derivative in order to reconstruct the acceleration and deceleration in a more realistic manner. From Equations (12)–(15), it can be seen that the control points can be computed by setting the parameter *u* to zero and one using the derivatives and equating it with the values defined by the input data. In our case, we had six conditions in total (Equations (11) plus the continuity of the second derivative at both ends). These lead to a quintic Bézier curve, the control points of which can be derived as:

(16) $$b_{0}=\left(t_{n},s_{n}\right),\;\;\;\;b_{5}=\left(t_{m},s_{m}\right)$$

(17) $$b_{1}=\left(t_{n},s_{n}\right)+\frac{1}{5}\left(1,v_{n}\right),\;\;\;\;b_{4}=\left(t_{m},s_{m}\right)-\frac{1}{5}\left(1,v_{m}\right)$$

(18) $$b_{2}=\left(t_{n},s_{n}\right)+\frac{2}{5}\left(1,v_{n}\right),\;\;\;\;b_{3}=\left(t_{m},s_{m}\right)-\frac{2}{5}\left(1,v_{m}\right)$$

**Figure 3:**
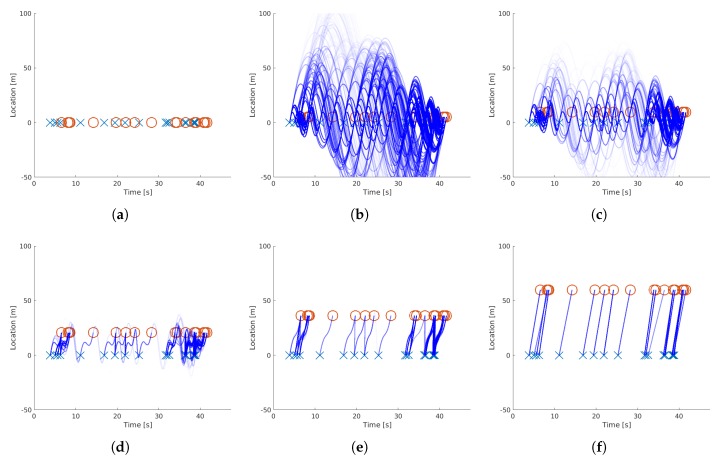
Iterative steps of the EM-algorithm with the visualization of the corresponding trajectories. The top left plot (**a**) shows the initial datasets, while the plots (**b**–**f**) are the visualizations of Iteration Steps 2, 4, 10, 24, and 100, respectively. In this figure, the opacity of the blue lines correspond to the results of the E-step, and the shift of the red circles corresponds to the M-Step of the EM approach, as described in Algorithm 1.

Figure 3 shows the iterative process of the proposed method. In the first image (a), the initial datasets are shown, where the crosses and circles are the timestamp and location entries of the first and second sensor, respectively. Note that all entries are at Location 0, as there is no prior knowledge of the location or time offsets between the sensors. In the second image (b), the Bézier curves of the matches are visualized. At the beginning of the algorithm, all pairs have similar matching probabilities, which is visualized by the similar opacity of the curves. The S-shape of the curve is originating from the condition of the speed continuity of the curve at the first and second detection with an unfinished spatial offset estimation. In the following plots, the spatio-temporal synchronization is shown by the shift of the circles (detections in the second sensor), while all the unrealistic curves get more transparent (less probable) until saturation. Please note that in this example, the reconstructed trajectory is the longitudinal one, so the resulting $s(t_{n})$ is the vehicle position along the road. As we will see in later sections, this work was conducted with the use of sensors that only deliver the timestamps of vehicles passing, without measuring the lateral position on the road. Nevertheless, the method can easily be applied to the lateral position in the same way in order to deliver lane changing maneuvers.

## 3. Experimental Results

In this section, we present the results of the behavior analysis of the described methods. In order to allow a comprehensive performance investigation, we used the different datasets consisting of synthetic, GNSS, and radar data. The validation steps applied were based on appropriate performance metrics for the problems presented in Section 2: matching, offset estimation, and trajectory reconstruction. The metrics will be discussed in more detail in this section.

### 3.1. Underlying Dataset

**Figure 4:**
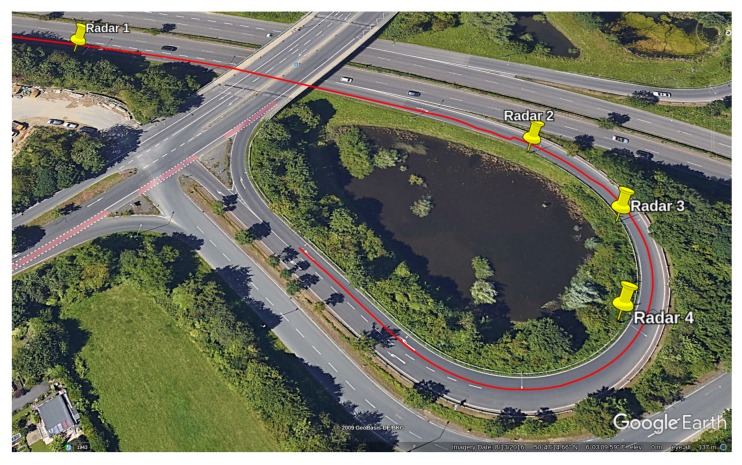
The location of the data recording. Four radar devices were installed at the poles marked here with yellow. The red line shows a path recorded with an RTK-GNSS sensor mounted on a vehicle.

We based our validation on data recorded on the expressway “Pariser Ring” in Aachen on 30 November 2018 (Figure 4). The experiment was conducted with the use of four radar devices that detected passing vehicles and recorded their timestamp and speed at the cross-section of the device. The distances between consecutive devices were measured and consisted of 155 m, 35 m, and 35 m, respectively.

It is important to note that the radar devices had separate internal clocks with limited synchronization possibilities. The resolution of the recorded timestamp was only one second, while the resolution of the speed was 1 km h^−1^. Another limitation of these devices is that they were installed and configured with a given angle to the street, and the exact cross-section of the detection was not known. We will analyze the effect of these limitations and also demonstrate that even under these circumstances, a reasonable reconstruction result is achievable.

### 3.2. Synthetic Data Validation

The validation based on synthetic data was mainly motivated by the possibility of a systematic performance analysis. First, if necessary, we can easily control the different sources of error of the recording devices. We can, for example, eliminate the quantization of the recording resolutions or errors in the detection location. This gives the possibility to analyze specifically the effect of each error source separately, which cannot be done with real data. Secondly, we can apply a preconfigured range for measurement errors (“synthetic errors”) in order to analyze specifically their effect on the results. Finally, the underlying microscopic vehicle data can also be generated and used as a perfect reference. In comparison, real reference data measured in vehicles can never be completely errorless.

We based the synthetic data generation on the measurements of the first radar device, as it recorded all passing vehicles on three lanes and thus delivered the largest and densest data volume. We extracted the data of the morning rush-hour and overcame the limitation of time resolution by adding a uniformly-distributed number of milliseconds between zero and 1000 to each recorded entry. We kept the recorded speed values and chose a second cross-section at a specified distance *d*. Then, for each vehicle, we generated a normally-distributed acceleration value with mean $m_{a}$ and standard deviation $\sigma_{a}$, and we calculated the time when the vehicle would reach the second sensor if it used the generated acceleration value. We then split the data, treating the start and end positions as the dataset of the first and second sensor accordingly. We of course neglected the location information from the datasets so that the original offset had to be reconstructed by our algorithm. In the different validation steps presented, we varied specific parameters like measurement errors and data volume. For each of the parameter values, we applied the EM algorithm a fixed number of times to have the mean and variance of the resulting output. We were also able to apply the algorithm with optimization in the space/time domain separately or with combined spatio-temporal optimization.

The considered performance metrics are defined as follows:

- **Offset reconstruction error**: the deviation between the sensor offset reference value and the reconstructed one. Depending on spatial or temporal optimization, this involves the location or time offset in meters or seconds.
- **Trajectory reconstruction error**: the deviation between reference vehicle trajectories and the reconstructed ones measured as a root-mean-squared error in meters.
- **Matching sensitivity**: the recall value between the correctly-matched pairs after reconstruction and the pair of the reference dataset.
- **Matching relevance**: the positive predictive value between the correctly-matched pairs after reconstruction and all the matched pairs.
- **Number of iterations**: the number of iterations the EM-algorithm requires to converge.

#### 3.2.1. Errorless Data

**Figure 5:**
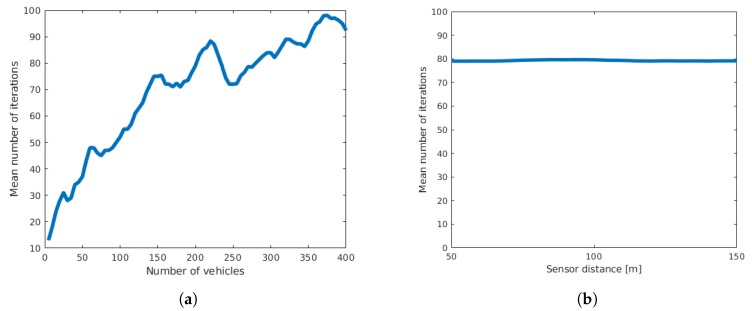
Number of iterations for algorithm convergence depending on (**a**) the number of vehicles and (**b**) the distance between sensors.

In this first validation step, we considered the case where the timestamp and speed of the vehicles can be recorded without error at the exact cross-section of the sensor. We varied the number of vehicles from 5–400 using a sensor distance of 100 m. Additionally, we varied the distance between the sensors from 50–150 m using 200 vehicles. We used 50 runs for each setting, resulting in a total of 4000 runs for the analysis of the vehicle count and 500 runs for the analysis of sensor distance. We first used only spatial optimization. The results showed that for all the settings, our algorithm was able to reconstruct both the sensor offsets and the vehicle trajectories perfectly, meaning that all appropriate metrics had zero mean. The only metric varying was the number of iterations for convergence, which seemed to depend on the number of vehicles used, but not on the distance between the sensors (Figure 5). We also validated the spatio-temporal estimation when we altered the sensor time offset by shifting each time entry of one dataset by a fixed value between one and 10 s and using a location offset of 100 m and a data volume of 200 vehicles. Just as in the case of the distance variation, the algorithm perfectly estimated both spatial and temporal offsets.

#### 3.2.2. Variance in Detection Location

Next, we analyzed the effect of varying the detection location. This constituted a type of error resulting from the fact that a sensor will not detect a vehicle at the exact same cross-section as it is installed, although we treated the data as such. In most cases, each vehicle will be detected at an individual cross-section depending on the physical characteristics, which influence the detection. An example would be the reflectance of the vehicle chassis in the case of a radar sensor, which will influence how early a vehicle can be detected. We used a total number of 200 simulated vehicle trajectories for this step, and 75 m, 100 m, and 125 m were used for the sensor distance. Note that we were still assuming that the detector made no error in speed measurement and started with the sensors being perfectly time-synchronized, so we only applied optimization in the space dimension.

**Figure 6:**
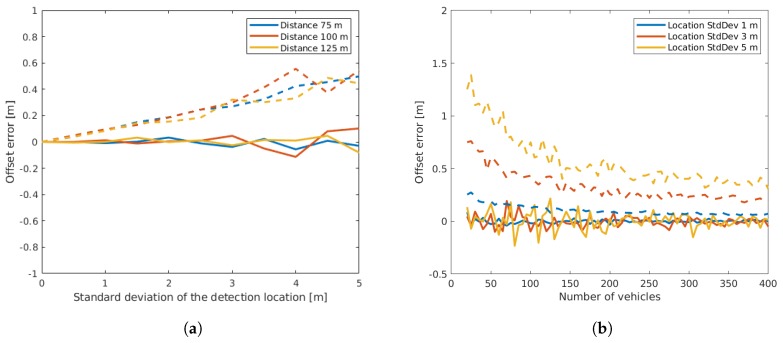
Offset estimation error depending on detection location (**a**) and depending on the number of vehicles (**b**). In both plots, the mean resulting error is plotted with a continuous line, while the standard deviation of the error is plotted with a dashed line.

**Figure 7:**
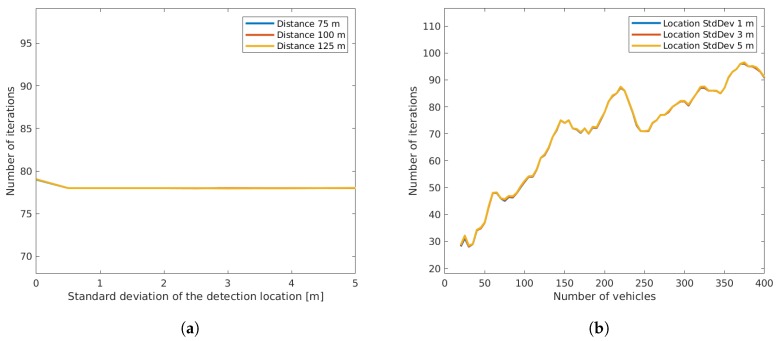
Number of iterations until convergence depending on detection location (**a**) and depending on the number of vehicles (**b**).

From Figure 6, it can be seen that the bias of the offset estimation error remained low, while a small variation was present due to the fact that a limited number of measurements (runs) had been used. Both results were independent of the sensor distance. The standard deviation of the estimation error increased linearly with the standard deviation of the detection location, but remained at a much lower level. This can be explained by the fact that the location errors of individual vehicles compensated each other. The right plot of Figure 6 shows that the standard deviation decreased with a higher number of vehicles. Thus, it is arguably sensible to use more vehicles for optimization to decrease the resulting error. On the other hand, a higher number of vehicles not only led to a higher number of required iterations (Figure 7), but additionally increased the computation time of each iteration as the dimensions of the matrices used in optimization increased.

**Figure 8:**
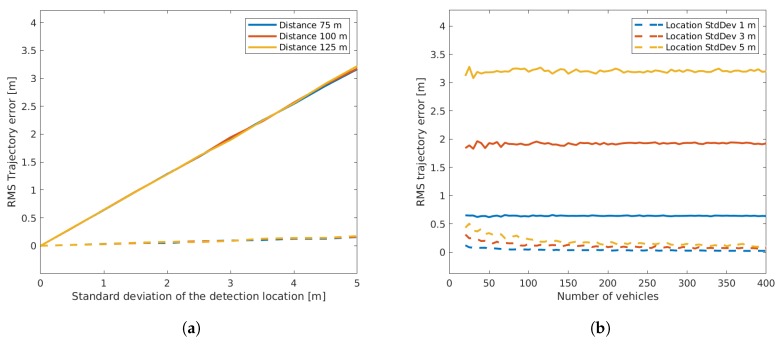
Trajectory RMS error depending on detection location (**a**) and depending on the number of vehicles (**b**). In both plots, the mean resulting error is plotted with a continuous line, while the standard deviation of the error is plotted with a dashed line.

In contrast to the sensor offset estimation, the mean RMS error of the vehicle trajectories only depended on the variance of the detection location and remained constant with increasing number of vehicles (see Figure 8). The linearity between the standard deviation in detection and the RMS error came from the relation between the end point of the computed trajectory and the calculated curve, which will adapt the location curve accordingly. The standard deviation of the mean RMS error showed a slightly negative slope, due to the variance of the mean being the variance of the individuals divided by the sample size, a property of independent identically distributed data. Thus, as the number of vehicles (sample size) became larger, the variance of the mean error decreased.

**Figure 9:**
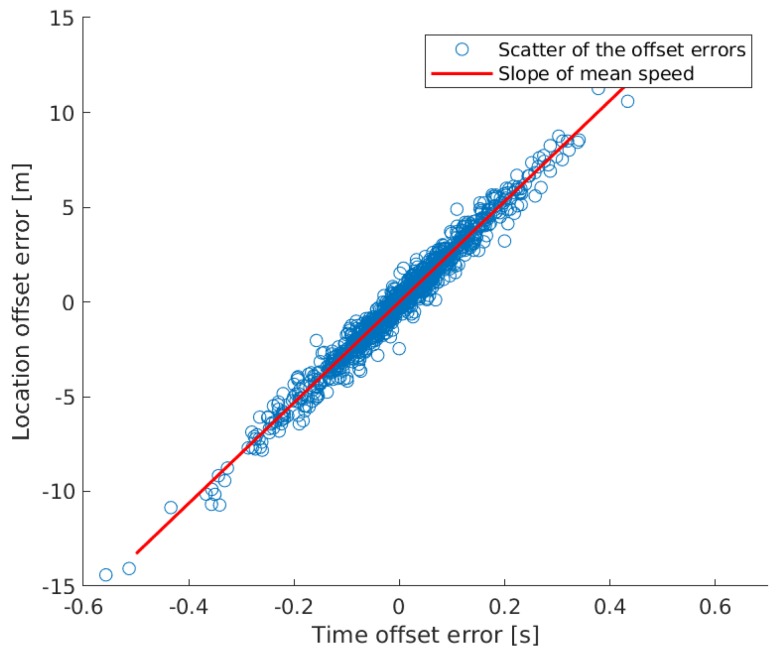
Scatter of the errors made in time and space offset estimation when using spatio-temporal optimization. The errors show a bivariate Gaussian distribution with the main axis representing the slope of the mean speed of the vehicles (red line).

We go on to examine the performance of the spatio-temporal offset estimation. Obtaining a time offset of zero is of course expected as we did not specifically add any time shift to the dataset. When looking at the results, the error values showed that while the time offset estimation mean was indeed at zero, the location estimation error significantly increased. Figure 9 shows a scatter plot between the time and space offset errors, where we observed that the values were distributed like a bivariate Gaussian, which seemed to be aligned with the mean speed of the vehicles. Indeed, deriving the eigenvectors of the covariance matrix showed that the principal axes of the Gaussian fitted to the data had a slope almost equal to the mean speed. Thus, when we only used the spatial optimization, the result was spread like a conditional Gaussian distribution at the vertical axes with the time offset of zero. The main reasoning here is that it is objectively reasonable to only use space optimization in order to significantly reduce errors in the estimation of location offset. In the following steps of validation, we will only consider the space optimization.

Because the performance showed very limited dependence on the sensor distance used, we will limit our further analysis to a distance of 100 m.

#### 3.2.3. Error in Speed Measurement

One other important source of error when recording traffic data consists of measuring the wrong speed of the vehicles. Although many of the vehicle detectors used in enforcement applications have a very high-quality standard and measurement accuracy, this error can never be completely neglected. Moreover, detectors developed for simpler applications like gathering traffic statistics most probably have lower accuracy standards. Thus, it is important to examine the influence of this error. In order to reduce the complexity of the results, we will neglect variations in the detection location. When not explicitly stated, the number of vehicles used for the optimization will be 200.

**Figure 10:**
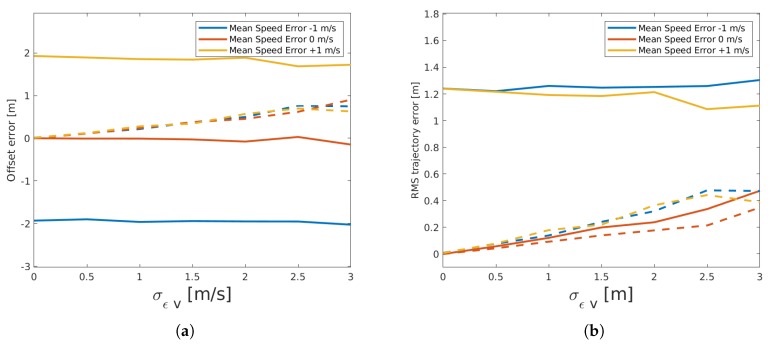
Influence of speed measurement error on the offset estimation (**a**) and on the trajectory RMS error (**b**). Continuous lines show the mean values, while dashed lines show the standard deviation of the error.

In the left plot of Figure 10, we show the results of the offset estimation after altering the measured vehicle speeds of the second sensor with a Gaussian noise, given its standard deviation. From this plot, we can see that the mean error of the offset estimation will only change with the mean error of the speed measurement. Additionally, the relationship between the values can be derived from the mean speed values of the vehicles, which was approximately 25 m/s. When changing the mean speed values of the second sensor, the slope of the linear trajectory used in the EM-algorithm changed to half that value. A distance between the sensors of 100 m led to 4 s of passing time, so the increase of the mean offset error would in this specific case be approximately double the value of the mean speed error. Similarly, the standard deviation of the resulting offset error only depended on the standard deviation of the speed measurement error, but not on the mean value. The right plot of the figure shows that the mean trajectory error changed linearly with $\sigma_{\epsilon v}$ when the mean speed error was zero. This is similar to what we see in Figure 8, although the mean RMS error was lower, while its standard deviation was higher. This means that a zero mean error in speed measurement will have a smaller influence on the result, but the error will be less predictable. In the case where the speed error had a mean value different from zero, the mean RMS error will be dominated by the mean measurement error rather than by its variance. Just as we have seen in the discussion of the detection location error, we can reduce the offset estimation error by increasing the number of vehicles. Naturally, this does not apply to the trajectory errors and will increase the number of iterations and the computation load.

#### 3.2.4. Quantization

Another limitation of the sensors we used was the level of quantization of the recorded values. Especially, the coarse resolution of time was a limiting factor when using our radar detectors, as they were only able to record timestamps up to a resolution of one second. This led to the necessity of examining the influence of the quantization on the results using our synthetic dataset. We conducted this experiment by again using 200 vehicles and three sensor distance values of 50 m, 100 m, and 150 m, as we expected that the distance could change the outcome. We neglected all previously-discussed errors by setting them to zero mean and variation. We applied a quantization to the time values of both sensors. We implemented the quantization by means of a factor value iterating from 1–10, which defined the partition of a second to which the recorded values were rounded. With a factor of 10, the values were rounded to a tenth of a second, while with a factor of one, the values were discretized to seconds just as the case in our real-world measurement.

**Figure 11:**
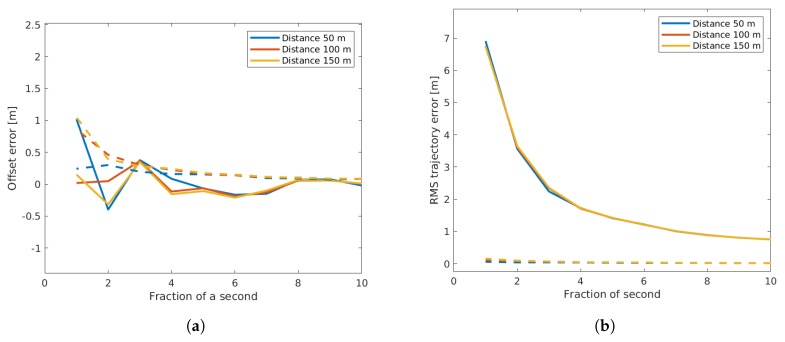
Influence of quantization on the offset estimation (**a**) and on the trajectory RMS error (**b**). Continuous lines show the mean values, while dashed lines show the standard deviation of the error.

As we can see from Figure 11, the quantization of the values did have a significant effect on both the sensor offset estimation and the trajectory reconstruction. Note that the lower values of the fraction of a second corresponded to a coarser quantization. As one would expect, the lower values not only led to a higher mean offset estimation error, but also to a higher standard deviation of this value. There was also some change due to the used distance, as a very coarse quantization led to higher errors when low distances were used. This can be explained by the fraction of the quantization time out of the complete passing time of the vehicles. If the quantization of the time values was in the magnitude of a second, then the slopes computed in the EM-algorithm were altered much more when short time ranges were used, thus leading to higher resulting errors. When we examine the outcome of the RMS trajectory error, we can clearly see that the errors in the trajectory reconstruction were a lot higher than in the offset estimation. This again can be explained by the compensation effect of using many different vehicles to estimate the sensor offset, while the errors of individual vehicles remained quite high. We can also see that the sensor distance did not affect the trajectory error. The other metrics, like the number of iterations and the matching sensitivity, were largely unaffected by quantization.

#### 3.2.5. Outliers

**Figure 12:**
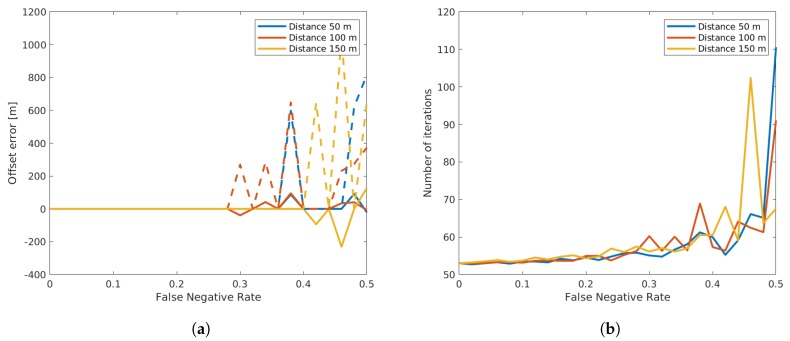
Influence of false negatives in the offset estimation (**a**) and on the number of required iterations (**b**). Continuous lines show the mean values, while dashed lines show the standard deviation of the result.

We have already discussed many possible measurement errors given a vehicle has correctly been detected. Nevertheless, depending on the circumstances of the recording and on the traffic density, the detectors will also record to some extent false negative and positive detections. For the presented algorithm, the false negatives of one sensor led to outliers in the dataset of the other sensor. Thus, in this validation step, we first examined how these outliers reduced the resulting accuracies. For the analysis, we varied a percentage of false negative detections for both sensors. We simply removed detections from both sensors at random accordingly to the false negative rate chosen. For each value, we again applied many runs with our algorithm, in each run generating new vehicle trajectories and deleting some of them as described. In Figure 12, we can see that the offset estimation error was zero up to a False-Negative-Rate (FNR) value of 0.25, after which the results became very unreliable. We must note here that in order to be able to examine the effect of outliers specifically, we neglected all other sources of error, which led to the estimation error value of zero. The number of required iterations also remained at a fairly low level up to the FNR value of 0.25.

**Figure 13:**
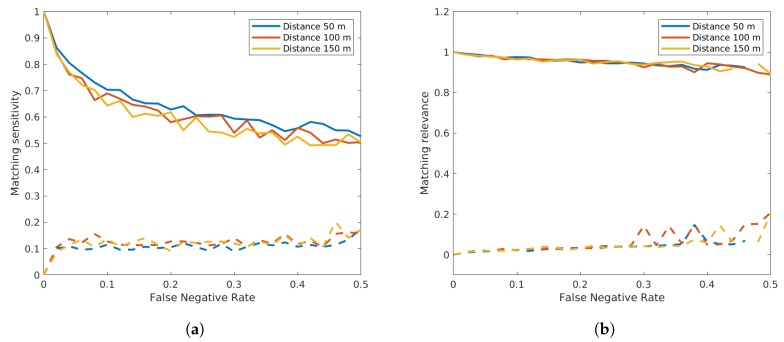
Influence of false negative detections on the matching sensitivity (**a**) and on the relevance (**b**). Continuous lines show the mean values, while dashed lines show the standard deviation of the result.

A very interesting effect can be seen in Figure 13, where the left plot shows that the matching sensitivity decreased quite fast. This means that out of all available matches still available in the dataset after deleting random vehicles, the algorithm can only find a portion of vehicle matches due to the very narrow Gaussian distribution after convergence. On the other hand, the right plot clearly shows that a very large portion of matches resulting from the algorithm were true matches, which explains the ability of precisely estimating the sensor offsets. This of course is a trade-off that can further be adapted. If we limit the convergence thresholds in the EM algorithm, one could achieve a vehicle registration sensitivity at the cost of more pairs of vehicles being falsely matched and consequently a higher offset estimation error.

**Figure 14:**
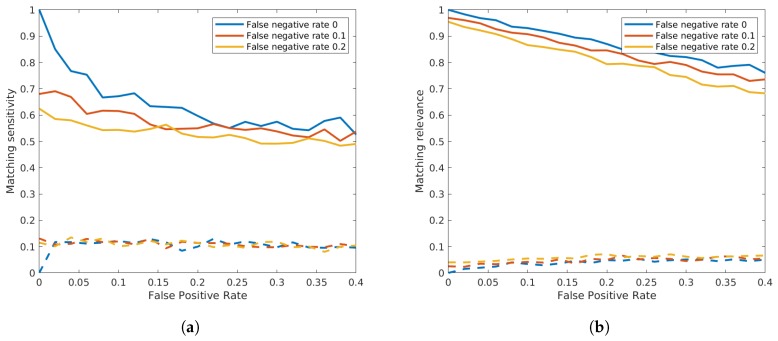
Influence of false positive detections on the matching sensitivity (**a**) and on the relevance (**b**). Continuous lines show the mean values, while dashed lines show the standard deviation of the result.

Similar to the false negative detections, we also simulated false positives. From the recorded radar data, it can be observed that false positive detections occurred mostly in the presence of vehicles. It did for example occasionally happen that a large vehicle like a bus or a truck was wrongfully detected as two small vehicles. In the absence of traffic, false positive detections were extremely rare. To simulate this fact, we specifically generated false positive detections at both sensors around the already existing detections. Thus, we extracted a number of vehicles according to the chosen false positive rate and copied the data of those vehicles also adding a random time to the original timestamp with a minimum and maximum absolute difference of 2 s and 3 s, respectively. In order to examine the combination between both false negatives and positives, we additionally set the false negative rate to 0, 0.1 and 0.2. The sensor distance was fixed to 100 m. Figure 14 shows that the matching relevance was significantly higher than the sensitivity, although the false positive rate seemed to have a slightly more negative effect on the resulting relevance than the false negative rate.

### 3.3. Real Data Experiment

**Figure 15:**
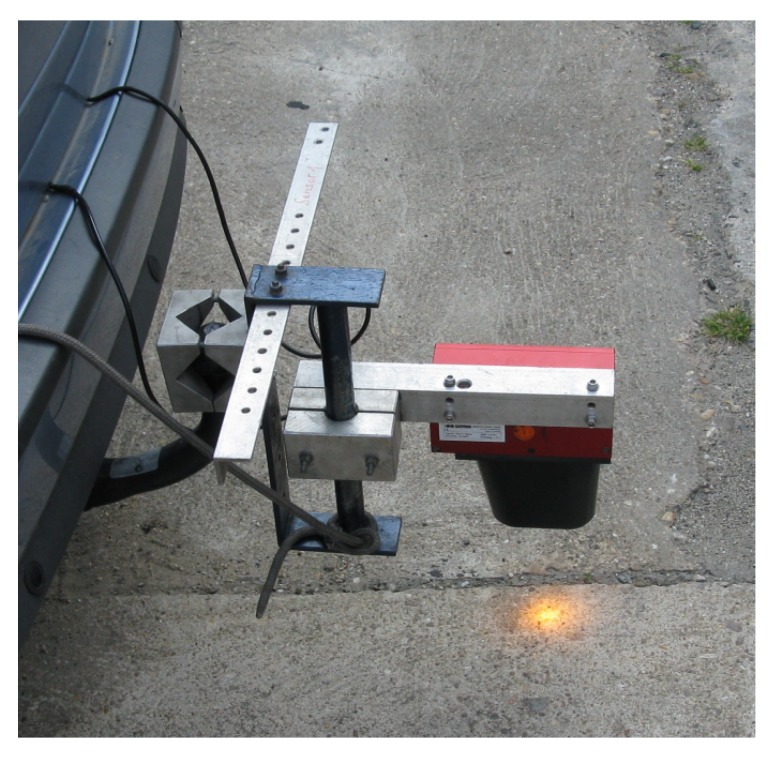
Laser Doppler velocimeter used to generate reference data for the validation of the trajectory reconstruction.

In addition to validation with synthetic data, in this subsection, we want to demonstrate the capabilities of the proposed methods using real measurements at two cross-sections. In order to be able to validate device offset reconstruction, we used high-precision RTK-GNSS sensors for two purposes: to record the exact location of the radar devices with a precision of a few centimeters and to record the vehicles’ paths along the curve. We were able to record the device cross-sections with a high precision with static GNSS data recording at the exact location of the radar devices. The GNSS data recorded when the sensor was mounted on the vehicle showed a high amount of jitter due to driving beneath the bridge seen in Figure 4, where satellite coverage was lost and could only be regained after 5–10 s, which limited our path reconstruction possibilities. Thus, instead of using the GNSS, our reference data of vehicle positions came from an optical distance measurement device mounted at the back of the vehicle (Figure 15). The sensor was based on laser Doppler velocimetry to measure the velocity and length of moving surfaces [28]. With the laser pointed downwards, the measured surface in this case was the road. The technique used had a very high accuracy, being capable of recording the distance and speed with an error less than ±0.1%. Additionally, the laser was coupled with a control box, which enabled recording the data with a connected notebook. On the other hand, the control box also provided the possibility of triggering a measurement reset either by a starter button or by an infrared light barrier. In the latter case, an additional laser device was directed sideways with reflecting markers being installed along the road. When the laser beam was reflected to the device, the measurement of the velocimeter was reset. We installed reflective markers at the location of the radar devices so that passing the relevant cross-sections triggered new distance measurements, and thus, we had the same coordinate system along the road section. At each new section between two radars, the record of the velocimeter started with zero.

For the validation of the reconstruction of microscopic traffic data, we drove with the equipped vehicle 33 times within three hours. For this period of time, we recorded both radar data and vehicle paths from the distance measurement device. As we saw in the previous subsection, our proposed algorithm was unable to converge to a good result when there were more than 25% outliers in the recorded data. When looking at Figure 4, we can see that the first radar device was located at a cross-section with three lanes, while the other devices only monitored the exit lane. This can also be verified in the radar data, as the first devices recorded 2928 vehicles in the same amount of time as the others recorded 444, 286, and 316 vehicles in the order of the devices. It is practically impossible to find feasible reconstruction between Radar 1 on the other devices. Thus, we validate the algorithm based on the data from Radar 2 and Radar 4. The distance between these two devices was 70 m along the curve, while all the devices were synchronized via Bluetooth using a handheld tablet, which connected to each radar device separately. The synchronization with the tablet was possible up to a 1-s deviation from the clock of the tablet. Additionally the radar devices were only capable of detecting vehicles with a resolution of 1 s.

**Figure 16:**
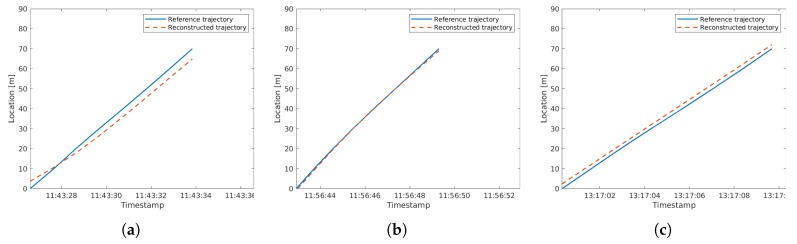
Longitudinal microscopic trajectory of the reference vehicle and the reconstructed data. The subplots (**a**–**c**) show three different vehicle passes with blue lines, while the red, dashed lines show the corresponging reconstructed trajectories (data recorded on the date of 30-November-2018).

We used the EM algorithm with both spatial and temporal optimization, as we could not guarantee a very precise time synchronization between the radar devices. The calculated offset after convergence was −0.63 s and 69.7 m between Radar 2 and 4, which matched very well with our measured reference. This indeed was in accordance with the results from the synthetic data validation. As the vehicles appeared randomly within the time windows of 1 s, the spatio-temporal offset error balanced out over many matched vehicle pairs. In other words, for a number of vehicle pairs that would correct the temporal offset towards the lower second value, their was a similar number of pairs that corrected the offset towards the higher second value. The results also delivered 274 matches between the two cross-sections. Moreover, from the 33 passes recorded with our equipped vehicle, we could successfully find 25 passes in the resulting matches. Figure 16 shows an outline of the reconstructed trajectories, where we can see that even with the limited accuracy and resolution specifications of the recording devices used, very good trajectory reconstruction was feasible. To be more precise, the resulting mean RMS trajectory error was 3.48 m with a standard deviation of 2.08 m over these 25 trajectories. This result also fit our expectations as the vehicle passes showed speeds of 10–14 m/s. With a quantization of 1 s, the detection time error for a vehicle could be at most 0.5 s (either rounding up or down), but the mean RMS time error was about 0.3 s, which led to the found RMS error for the mentioned speed values.

## 4. Conclusions

This paper presented a method for spatio-temporal synchronization and microscopic traffic data reconstruction from cross-section-based detection devices. The required input data consisted of the timestamp and speed of individual vehicles. The three main problems that were been overcome were: unknown offset of the recording devices, unknown vehicle matches, and unknown vehicle trajectories. The proposed methods were based on the assumption of limited vehicle dynamics between the recording devices. It has been shown how under these assumptions, the iterative EM-algorithm can be used to register the individual detections of the devices and estimate the previously unknown offset simultaneously. In a further step, the registered datasets were used to reconstruct trajectory data between the devices by means of quintic Beziér curves.

After presenting the basic methodology, a comprehensive validation was presented to demonstrate the capabilities of the method. Using synthetically-generated datasets, different error possibilities, like location variance, speed measurement error, and outliers in the input data, were discussed, and their effect on the outcome of the method was thoroughly investigated. It was shown that errors in the offset estimation can be reduced by increasing the number of vehicles in the datasets, which led to a higher computational load. Thus, one must find a trade-off between these two parameters. Additionally, on the synthetic datasets, a validation based on empirical data was also conducted, where was shown that the algorithm not only precisely estimated the offsets of the used devices, but also accurately reconstructed vehicle trajectories.

Based on this work, further validation of the matching performance could be conducted by precisely recording individual vehicle matches. This could be done by using detectors based on number plate recognition. Furthermore, the applicability of the proposed methods should also be demonstrated by using other types of cross-section detectors like inductive loops and computer vision systems. In order to provide more information of the vehicle passes, the method could be applied in a multidimensional space to also reconstruct the lateral movements of vehicles rather than only longitudinal trajectories. Additionally, an iterative version of the method could be developed to be able to optimize continuously the parameters of the cross-sections with a continuously-growing data volume. Finally, a very interesting and promising extension of this work would be a thorough analysis on the possibility of overcoming the limitations regarding dynamic behavior. If the propagation of shock waves in congested traffic is of interest, many dynamic maneuvers between cross-sections need to be taken into account. This could be done by incorporating driver modeling and microscopic traffic simulation in combination with data on the vehicle lengths in order to derive complex vehicle interactions.

## Abbreviations

The following abbreviations are used in this manuscript:

V2V	Vehicle-to-Vehicle
V2I	Vehicle-to-Infrastructure
EM	Expectation Maximization
RTK-GNSS	Real-Time Kinematic Global Navigation Satellite System
FNR	False-Negative-Rate
RMS	Root Mean Square
